# Fine mapping and breeding application of two brown planthopper resistance genes derived from landrace rice

**DOI:** 10.1371/journal.pone.0297945

**Published:** 2024-04-16

**Authors:** Fahuo Li, Liuhui Yan, Juan Shen, Shuolei Liao, Xianrong Ren, Ling Cheng, Yong li, Yongfu Qiu

**Affiliations:** 1 College of Agriculture, Guangxi Key Laboratory of Agro-environment and Agric-Products Safety, State Key Laboratory for Conservation and Utilization of Subtropical Agro-bioresources, Guangxi University, Nanning, China; 2 Guangxi Key Laboratory of Crop Cultivation and Physiology, Education Department of Guangxi Zhuang Autonomous Region, Nanning, China; 3 Liuzhou Branch, Guangxi Academy of Agricultural Sciences, Liuzhou Research Center of Agricultural Sciences, Liuzhou, China; 4 College of Agriculture, Yangtze University, Jingzhou, China; University of Agricultural Sciences Raichur, INDIA

## Abstract

The Brown planthopper (*Nilaparvata lugens* Stål; BPH) is known to cause significant damage to rice crops in Asia, and the use of host-resistant varieties is an effective and environmentally friendly approach for controlling BPH. However, genes limited resistance genes that are used in insect-resistant rice breeding programs, and landrace rice varieties are materials resources that carry rich and versatile genes for BPH resistance. Two landrace indica rice accessions, CL45 and CL48, are highly resistant to BPH and show obvious antibiosis against BPH. A novel resistance locus linked to markers 12M16.983 and 12M19.042 was identified, mapped to chromosome 12 in CL45, and designated *Bph46*. It was finely mapped to an interval of 480 kb and *Gene 3* may be the resistance gene. Another resistance locus linked to markers RM26567 and 11MA104 was identified and mapped to chromosome 11 in CL48 and designated *qBph11*.*3* according to the nominating rule. It was finely mapped to an interval of 145 kb, and *LOC_Os11g29090* and *LOC_Os11g29110* may be the resistance genes. Moreover, two markers, 12M16.983 and 11MA104, were developed for CL45 and CL48, respectively, using marker-assisted selection (MAS) and were confirmed by backcrossing individuals and phenotypic detection. Interestingly, we found that the black glume color is closely linked to the BPH resistance gene in CL48 and can effectively assist in the identification of positive individuals for breeding. Finally, several near-isogenic lines with a 9311 or KW genetic background, as well as pyramid lines with two resistance parents, were developed using MAS and exhibited significantly high resistance against BPHs.

## 1. Introduction

The brown planthopper (*Nilaparvata lugens* Stål; BPH) is a pest that causes extensive damage to rice crops across Asia. Its presence can lead to rice plant mortality due to its habit of residing in rice stems and sucking sap. Additionally, they can spread diseases, resulting in reduced yield or crop failure [1]. Although pesticides are often used to prevent and control BPH, they only provide short-term effectiveness and have significant drawbacks, including poor sustained effects, high costs, and environmental pollution. Moreover, heavy reliance on pesticides can lead to BPH resistance and a resurgence in populations [2]. Identifying major resistance genes and cultivating resistant varieties are considered the most effective approaches for managing BPH. In addition to wild rice, many landrace rice accessions have excellent agronomic traits and carry rich resistance genes essential for rice resource diversity. Therefore, there is an urgent need to identify, map, and clone more resistance genes to understand the mechanisms of resistance in rice.

Since the 1960s, different rice varieties have yielded 45 BPH resistance genes clustered on several rice chromosomes. For instance, chromosomes 4, 12, 6, and 3 contain at least 20, 9, 7, and 6 BPH resistance genes, respectively. Four categories of BPH-resistance genes were cloned and differentiated based on their protein sequences. The first category comprises plasma membrane protein pattern recognition receptors (PRRs), which constitute the first layer of the rice immune system. They are activated by conserved herbivore-associated molecular patterns (HAMP) [3], including *Bph3* on chromosome 6 [4] and *Bph15* on chromosome 4 [5]. The next category encodes intracellular nucleotide-binding domains and leucine-rich repeats (NLR), which sense effectors sent to rice cells and trigger the corresponding defense response. It includes *Bph14* on chromosome 3 [6]; *Bph6*, *Bph30*, and *Bph40* on chromosome 4 [7, 8]; *Bph37* on chromosome 6 [9]; and *Bph9*, *Bph18*, and *Bph26* on chromosome 12 [10–12]. The third type encodes a protein with a B3 DNA-binding domain, such as *bph29*, located on chromosome 6 [13]. The final category encodes proteins containing a short consensus repeat (SCR) domain, such as *Bph32*, on chromosome 6 [14].

Marker-assisted selection (MAS) is an effective molecular breeding technique widely used in breeding programs for BPH resistance. For instance, Hu et al. introduced *Bph14* and *Bph15* into the rice line Zhenshan97B using MAS technology to enhance its resistance to BPH [15]. Liu et al. (2016) introduced two BPH resistance genes, *Bph3* and *Bph27(t)*, into the susceptible variety, Ningjing 3, using MAS technology, which significantly improved BPH resistance [16]. Li et al. (2019) introduced four BPH resistance genes, *Bph36*, *Bph27(t)*, *bph29*, and *Bph3*, into rice lines BR1658 and BR1660 using MAS technology and obtained high-resistance lines [17].

In the present study, we aimed to explore the resistance characteristics and map the BPH resistance genes of two highly resistant landrace rice accessions, CL45 and CL48. Several near-isogenic lines (NILs) and pyramid lines (PYLs) carrying BPH resistance genes have been developed using MAS. The mapped resistance genes and the developed resistance lines can contribute to insect-resistant rice breeding programs.

## 2. Materials and methods

### 2.1. Materials, mapping populations, and insects

Landrace rice accessions CL45 and CL48 were collected from Guangxi, China, and surveyed for BPH resistance at the seedling and tillering stage (S1A–S1H Fig). These cells showed high resistance to BPH and were used as resistant parents. Indica rice lines 9311 and KW exhibited comprehensive merit traits and were highly susceptible to BPH. These were used as recipient parents or susceptible controls.

To develop the mapping population, CL45 and CL48 were crossed with 9311 and KW, respectively, and were self-crossed twice to develop two F_3_ populations. One population derived from 9311/CL45 contained 123 lines; the other, KW/CL48, contained 101 lines (S2 Fig).

To develop resistant NILs, the positive F_1_ individuals derived from 9311/CL45 and KW/CL48 were backcrossed four times with the recurrent parent 9311 or KW and self-crossed three times to obtain the BC_4_F_4_ population. PYLs were developed by crossing two resistant parents, CL45 and CL48. F_1_ individuals were continuously self-pollinated five times to produce the F_6_ generation, from which the plants homozygous for both resistance genes were selected (S2 Fig). The MAS method was used to detect the positive individuals in each generation.

Normally, germinating seeds were planted in plastic cups, pots, or buckets. The seedlings of parents (CL45, CL48, 9311, and KW) and F_3_ lines (9311/CL45 and KW/CL48) grew under average temperatures of 26–32°C and relative humidity of 75% with natural light.

BPHs were collected from a natural population of rice fields in Nanning, Guangxi, and the race of BPH was biotype II that the predominant biotype was detected in most of the rice-growing regions in China [18]. BPHs were subsequently propagated using a susceptible line 9311 in a greenhouse, with a temperature of 26–32°C and relative humidity of 75%.

### 2.2. BPH resistance evaluation

Several tests were conducted to explore the resistance characteristics of CL45 and CL48, including the BPH host choice, BPH survival, BPH body weight gain, and honeydew excretion weight test [19–21]. For the host choice test, two seedlings (12 days old) of one resistant and one susceptible parent were transplanted diagonally into a plastic bucket (d = 29 cm, h = 20 cm) for 30 days. The two seedlings types were infested with 20 BPH nymphs and subsequently covered with a fine light-transmitting mesh. The number of BPH settled on each plant was counted at 3, 6, 9, 24, 48, 72, 96, and 120 h post-infestation across eight replicates. For the BPH survival test, each seedling (12 d old) was transplanted into a plastic cup (d = 29 cm, h = 20 cm) for 30 days, where each seedling was infested with 10 BPH nymphs and covered with a plastic cup. The surviving BPH in each plant was recorded daily for nine days across the eight replicates. For BPH body weight gain and honeydew secretion weight test, seedlings (12 d old) were transplanted into plastic buckets (d = 35 cm, h = 20 cm) and grown for 30 days. The parafilm wax bag (l = 3.5 cm, w = 3 cm) was weighed and fastened to the rice stem. BPH were weighed and placed in a wax bag for 48 h, then removed and weighed again to calculate weight gain, while the wax bag was weighed again to measure honeydew secretion weight. Twenty wax bags were used for testing.

A seedling bulk test was performed as previously described to assess BPH resistance of the developed F_3_ populations [19]. A total of 123 lines from the 9311/CL45 F_2:3_ population and 101 lines from the KW/CL48 F_2:3_ population were used. Seeds of the parent and F_3_ lines were sown in separate plastic trays (l = 58 cm, w = 38 cm, and h = 9 cm), with 20 plants per row and a total of 30 rows per tray. The seedlings (12 days old) were infested with approximately eight BPH nymphs per seedling. When all susceptible parent seedlings died (scored as 9), each seedling was given a score of 0, 1, 3, 5, 7, or 9 according to the seedling bulk test scoring standard. The resistance score of each row was the weighted average score of each seedling. The evaluation experiments were repeated three times. The scoring standard is divided into six grades. Grade 0, healthy plant growth without leaf damage; grade 1, 1 yellow leaf; grade 3, 1 to 2 yellow leaves, or 1 withered leaf; grade 5, 2 to 3 yellow leaves, or 2 withered; grade 7, 3 to 4 yellow leaves, or 2 withered; and grade 9, total death [20, 21].

### 2.3. Gene mapping

Bulk segregation analysis (BSA) was used to examine molecular markers and explore the resistance loci in the 9311/CL45 and KW/CL48 populations [22]. Ten extreme resistance plants derived from the F_2_ population were selected, and the genomic DNA of each was extracted and diluted to 100 ng/μL; then, 20 μL DNA of each sample were mixed to develop one resistance pool. The same method was used to develop the susceptible DNA pool. A total of 1,560 simple sequence repeat (SSR) and insertion-deletion (InDel) markers were screened to select polymorphic primers between the parents. Polymorphic markers were detected in the two extreme DNA pools. Furthermore, more polymorphic markers were identified between the parents in the target chromosomal region. In the F_2_ populations, the bands consistent with the resistant parents (CL45 and CL48) or susceptible parents (9311 and KW) were designated to be B and A, respectively; the samples having both bands of resistant and susceptible parents were designated to be H. JoinMap 3.0 was used to develop the local genetic linkage map, and MapQTL 5 was used to detect the resistance locus of the F_2_ population by analyzing phenotype and genotype data [23, 24]. It indicates one resistance locus in the mapping population when the threshold largest limit of detection (LOD) value is ≥ 3.0.

Based on the interval position of QTL localization, we selected markers on both sides of the localization to screen for individual recombinant plants in the F_3_ or BC_1_F_3_ populations, as well as to identify the phenotype of the recombinant individuals with BPH, develop new primers, and obtain the genotypes of these recombinant individuals using the newly developed primers.

Primer development was aimed at specific QTL localization intervals using BLAST tools from websites such as the National Center for Biotechnology Information (NCBI), Rice Genome Annotation Project (RGAP), and Rice Information GateWay (RIGW). By comparing the genomic sequences of Nipponbare (NIP), MingHui63 (MH63), 9311, and ZhenShan97 (ZS97), primers with product sizes between 100–300 bp were designed using the Premier Primer software (version 5.0) for insertion and deletion regions of over 10 bp. SSR molecular marker primers were obtained from the GRAMENE database (http://archive.gramene.org/markers/microsat/). The primer BLAST tool in NCBI was used to compare the designed primers, and primers with high specificity were selected as candidates and synthesized by Huada Gene Co., Ltd.

### 2.4. The expression of candidate gene

We performed qRT-PCR to explore the expression of candidate genes after BPH infestation in resistant and susceptible rice accessions. Seeds of the four rice accessions (CL48, CL45, 9311, and KW) were sown in plastic cups (d = 17 cm, h = 15 cm) with eight plants per cup and grown for 12 days. Each cup of rice was infected with 64 nymphs of BPH, which were infected at four time points: 48, 24, 12, and 0 h (without infection). The seedlings were infected at different time points and sampled together at the same time. Leaf-sheath was collected quickly for total RNA extraction (Takara TransZol Up Plus RNA Kit) and cDNA preparation (PrimeScript™ First-Strand cDNA Synthesis SuperMix kit) at 0, 12, 24, and 48 h post-infestation, and three replicates were carried out at each treatment time point. CL45 and CL48 samples without BPH treatment were used to sequence the coding sequences (CDS) of the candidate genes.

### 2.5. Statistical analyses

One-way ANOVA was used to compare multiple samples, and the t-test was used to examine the differences between the two groups. All the data were compared using the least significant difference (LSD) test at the 5% or 1% significance level.

## 3. Results

### 3.1. Landrace rice accessions CL45 and CL48 showed high resistance to BPH

**Fig 1:**
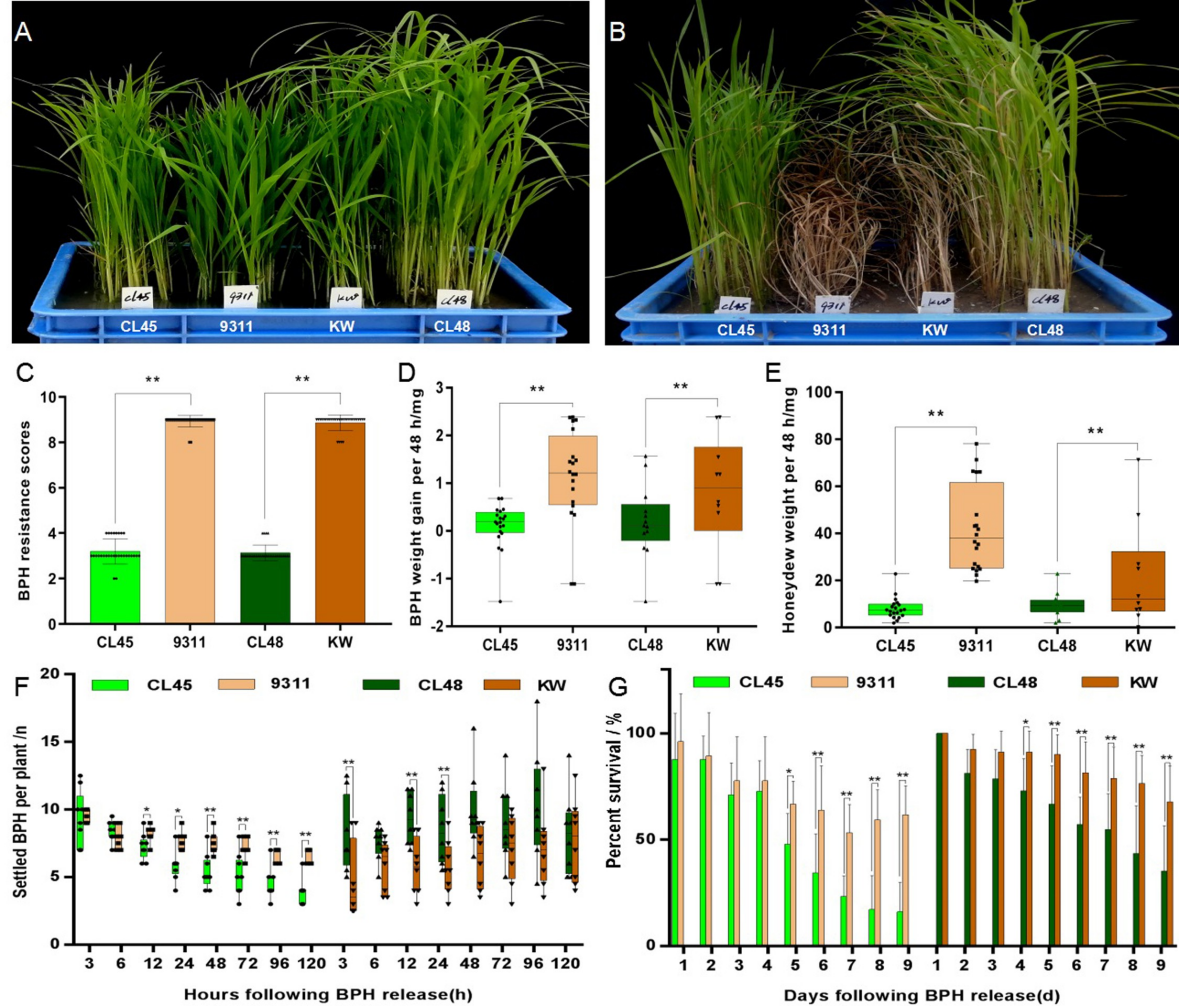
BPH resistance evaluation of rice accessions CL45 and CL48. (A, B) Resistance detection at the seedling stage before and after infestation. (C) BPH resistance scores at the seedling stage. (D, E) Detection of BPH body weight gain and honeydew excretion weight after 48 h treatment. (F) Detection of BPH host choice test. (G) Detection of BPH survival rates. Bars represent means of nine replicates for (A, B, C), twenty replicates for (D, E), and eight replicates for (F, G). Error bars represent the standard deviation (SD). *, P < 0.05; **, P < 0.01.

CL45 and CL48 exhibited a typical green color with mild damage symptoms, whereas 9311 and KW showed whole leaf wilt and death after 14 days of BPH infestation at the seedling stage (Fig 1A–1C) [20, 21]. The average body weight gain of one BPH was 0.23 and 0.19 mg fed on CL45 and CL48 plants, respectively, but more than 0.80 mg when fed on 9311 and KW (Fig 1D). Meanwhile, the honeydew weight excreted by one single BPH was 10.32 and 9.54 mg on CL45 and CL48 plants, respectively, but it was more than 20 mg on 9311 and KW (Fig 1E). The number of BPH distributed on 9311 plants was significantly higher than that of CL45 at all the observation time points, except at 3 and 6 h after infestation, whereas it was significantly higher in CL48 than in KW at the observation time points of 3, 12, and 24 h (Fig 1F). The BPH survival rates of CL45 and CL48 were significantly lower than those of the susceptible parents after four days of infestation (Fig 1G). These results indicated that BPH growth and development were inhibited in CL45 and CL48 plants.

### 3.2. BPH resistance evaluation and gene identification of mapping populations

**Fig 2:**
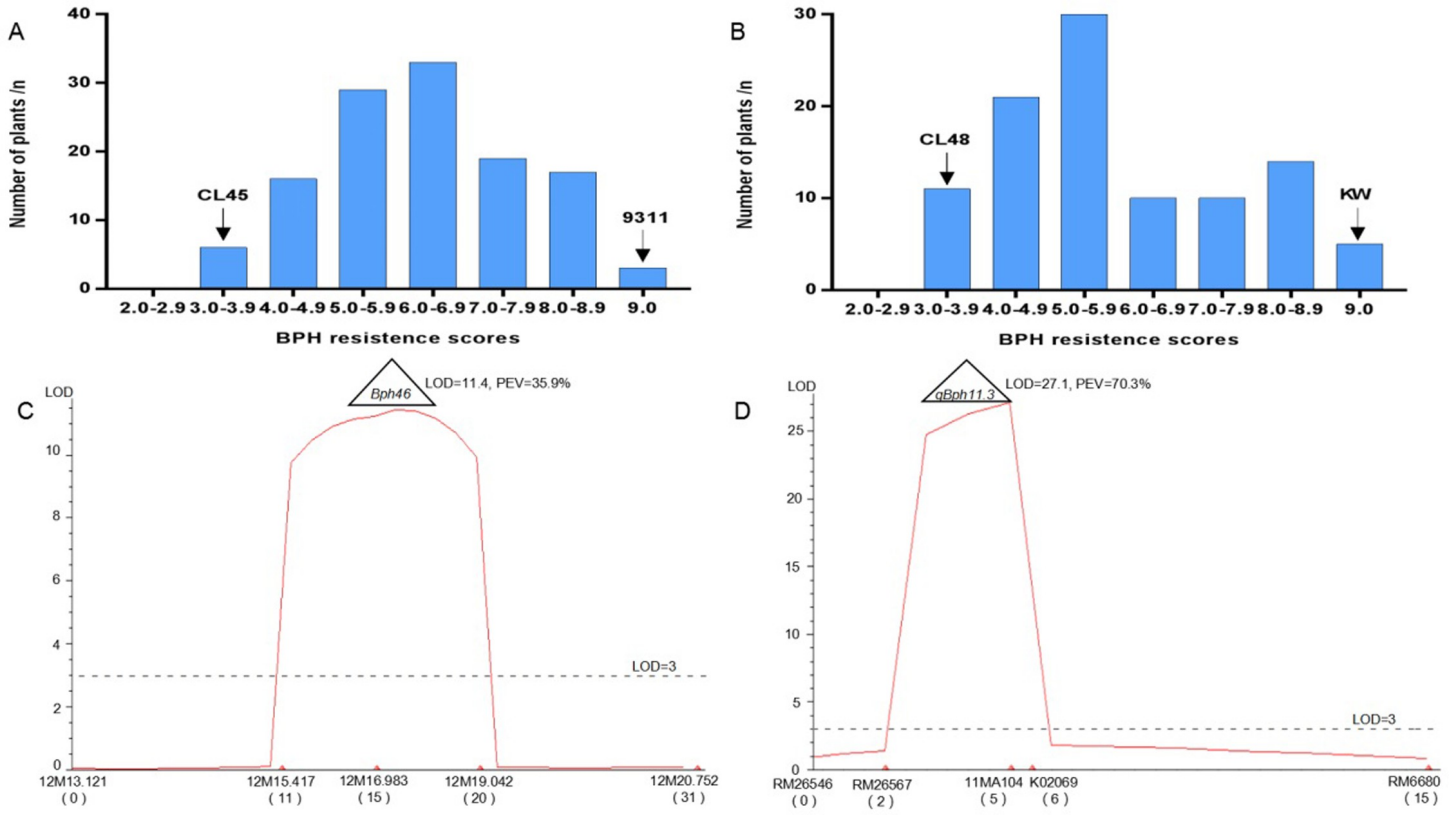
BPH resistance phenotypes and resistance gene/QTL were detected in the mapping population. (A) Resistance scores of mapping population derived from 9311/CL45. (B) Resistance scores of mapping population derived from KW/CL48. (C) BPH resistance gene/QTL detection using mapping population of 9311/CL45. (D) BPH resistance gene/QTL detection using mapping population of KW/CL48. Markers are given along the X-axis and LOD scores are shown on the Y-axis. Horizontal dashed line LOD score = 3, PEV phenotypic variance explained by the locus.

To identify the resistance genes in CL45 and CL48, a seedling bulk test was performed to evaluate the phenotypes of the mapped populations. As a result, the resistance scores showed continuous distribution and ranged from 2.5 to 9.0 for both F_3_ populations (Fig 2A and 2B). Based on the resistance evaluation criteria and a previous study, lines were divided into R (0–3.9), MR (4.0–6.9), and S (7.0–9.0) according to their resistance scores [19, 20, 25]. Consequently, the segregation of the resistant (R and MR) to susceptible (S) lines was found to be 84:39 for the population of 9311/CL45 (χ^2^ = 2.95 < χ^2^_0.01,1_ = 3.84) and 72:29 for the population of KW/CL48 (χ^2^ = 0.74 < χ^2^_0.01,1_ = 3.84). These results indicate that a single dominant gene in the CL45 and CL48 populations most likely controls BPH resistance.

To identify the resistance genes of CL45 and CL48, 422 markers were found to be polymorphic between CL45 and 9311, among which only three markers, 12M13.121, 12M16.983, and 12M19.042, on chromosome 12, were polymorphic between the resistant and susceptible DNA pools. A total of 449 markers were found to be polymorphic between CL48 and KW, and only markers RM26567, 11MA104, and K02069 on chromosome 11 were polymorphic between the resistant and susceptible DNA pools. Furthermore, two more polymorphic markers between parents for the populations of 9311/CL45 and KW/CL48 were obtained from the regions of interest, and the genotypes of all markers were surveyed in the F_2_ populations. Finally, one locus with the largest LOD value (11.4) was detected in CL45 between markers 12M16.983 and 12M19.042, explaining 35.9% of the phenotypic variation in the mapped population (Fig 2C). This was designated as *Bph46*, as no BPH resistance genes were reported in the mapping region. Similarly, one locus with the largest LOD score (27.1) was detected in CL48 between markers RM26567 and 11MA104, explaining 70.3% of the phenotypic variation in resistance (Fig 2D). It was tentatively designated *qBph11*.*3* according to the nomination rule.

### 3.3. Fine mapping of *Bph46* derived from CL45

**Fig 3:**
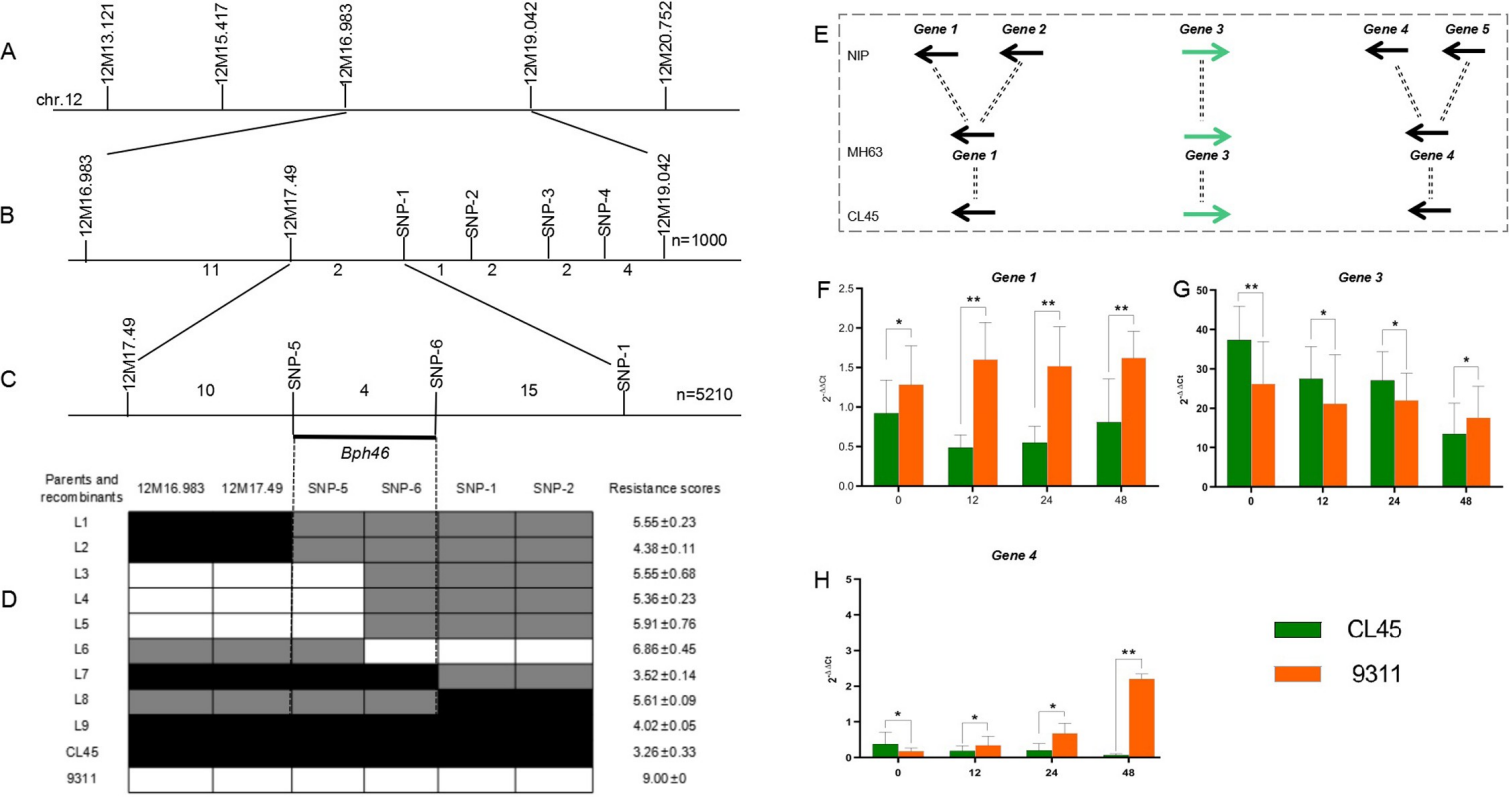
Fine mapping of *Bph46*. (A, B, C) Markers correspond to specific locations on chromosome 12. N denotes recombinants. The numbers below the line in (B, C) indicate recombinants between adjacent markers; (D) Genotypes and phenotypes of the selected recombinants. Black, white, and gray bars denote the resistant parent homozygotes CL45, susceptible parent homozygotes 9311, and heterozygotes, respectively. L1–L9 means recombinants derived from the generations of 9311/CL45. BPH resistance score is given as the mean value ± SD (n = six replicates). (E) Distribution of candidate genes in NIP, MH63, ZS97, and CL45. (F, G, H) qRT-PCR validation of the candidate genes in CL45 and 9311. OsAction1 was used as an internal control. The relative expression level of each gene was measured using the 2^-△Ct^ method. Bars represent the means of three replicates. Error bars represent the SD. *, P < 0.05; **, P < 0.01.

To finely map the resistance genes, we identified 22 recombinants derived from 1000 F_3_ seedlings of 9311/CL45 using the markers 12M16.983 and 12M19.042. We also developed one polymorphic SSR marker, 12M17.49 and four polymorphic SNP markers (SNP-1, SNP-2, SNP-3, and SNP-4) in the target region (Fig 3A and 3B). Two recombinants, L1 and L2, could determine the left marker 12M17.49, and the other two recombinants, L7 and L8, could determine the right marker, SNP-1, of the mapping region. Therefore, *Bph46* was mapped to one region flanked by markers 12M17.49 and SNP-1 (Fig 3C and 3D).

To obtain a high-resolution map of *Bph46*, we screened additional recombinants from the F_3_ population and identified two polymorphic SNP markers, SNP-5 and SNP-6, in the target region (Fig 3C). Three recombinants, L3, L4, and L5, could determine the left marker, SNP-5, and L6 could choose the right marker, SNP-6, in the target region (Fig 3D). It was finally mapped to be one region harbored by markers SNP-5 and SNP-6, which was approximately 480 kb according to the reference genome of the NIP. A total of 78 genes were predicted in the target region, and only five disease-resistance genes were detected in the NIP genome (S1 Table).

To analyze candidate genes, we sequenced five disease-resistance genes from CL45 and identified three resistance genes in CL45 and MH63 (Fig 3E). We conducted qRT-PCR to validate the three disease-resistance genes in CL45 and 9311. It can be seen that *Gene 1* and *Gene 4* in CL45 showed lower gene expression than that of 9311 after BPH treatment (Fig 3F and 3H). The expression level of *Gene 3* was higher in CL45 than in 9311 at 0 h, 12 h, and 24 h, while it was the opposite at 48 h (Fig 3G). These results suggested that *Gene 3* may be involved in the resistance of CL45. We further sequenced the CDS of *Gene 3* from CL45. Consequently, one 6-bp deletion in 5’-UTR and eleven SNP changes in CDS of *Gene 3* were detected when compared to MH63 (S3 Fig).

### 3.4. Fine mapping of *qBph11*.*3* and analyzing the candidate genes in CL48

**Fig 4:**
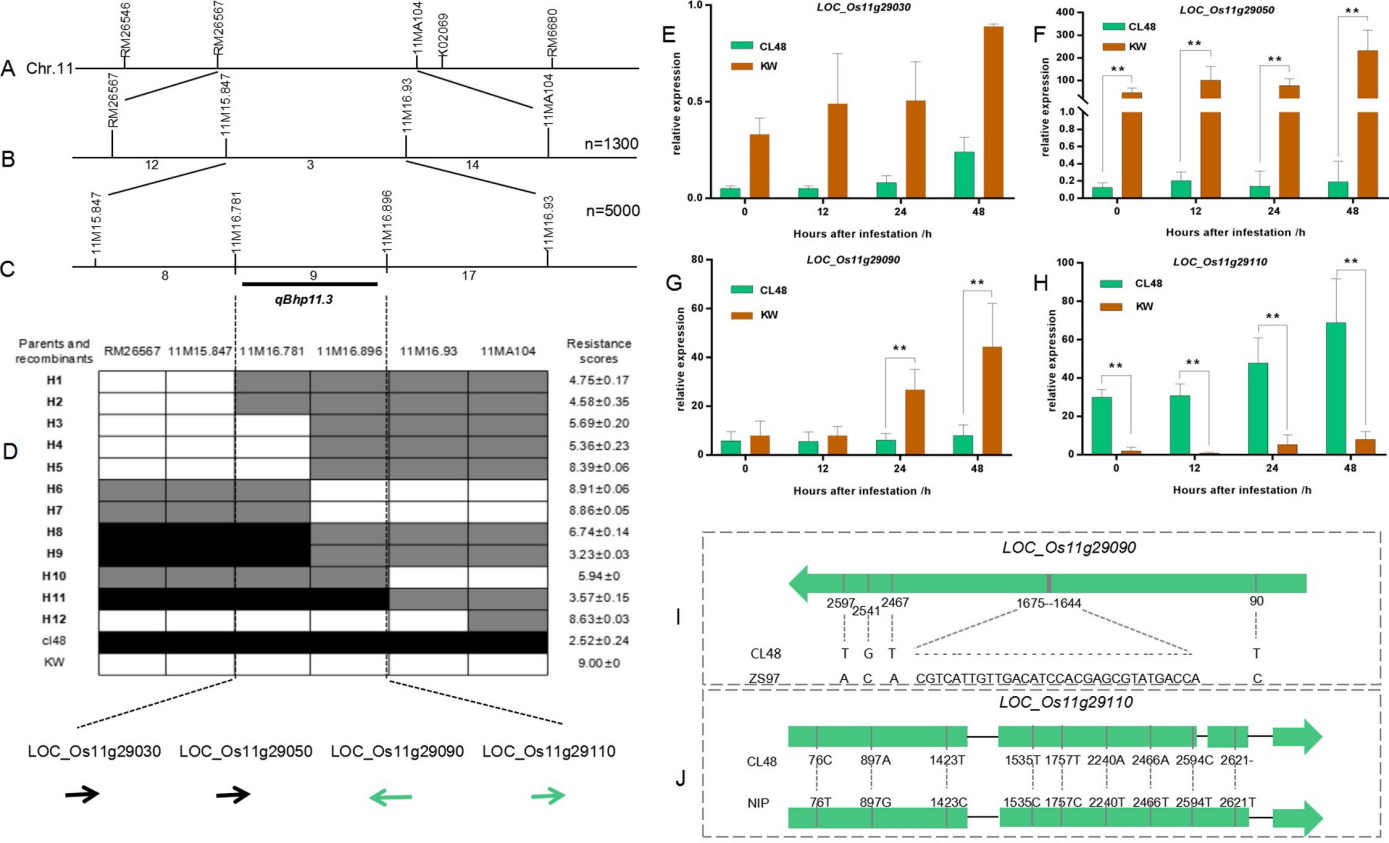
Fine mapping and candidate genes analysis of *qBph11*.*3*. (A, B, C) Markers correspond to specific locations on chromosome 11. N denotes recombinants. The numbers below the line in (B, C) indicate recombinants between adjacent markers; (D) Genotypes and phenotypes of the selected recombinants. Black, white, and gray bars denote the resistant parent homozygote of CL48, susceptible parent homozygote of KW, and heterozygotes, respectively. H1–H11 means recombinants derived from the generations of KW/CL48. BPH resistance score is given as the mean value ± SD (n = six replicates). (E, F, G, H) qRT-PCR validation of the candidate genes in CL48 and KW. OsAction1 was used as an internal control. The relative expression level of each gene was measured using the 2^-△Ct^ method. Bars represent the means of three replicates. Error bars represent the SD. *, P < 0.05; **, P < 0.01. (I) CDS comparison of *LOC_Os11g29090* between CL48 and ZH97. (J) CDS comparison of *LOC_Os11g29110* between CL48 and NIP.

To fine-map the resistance gene, we identified 29 recombinants derived from 1,300 F_3_ seedlings of KW/CL48 using markers RM26567 and 11MA104 and developed two polymorphic InDel markers, 11M15.847 and 11M16.93, in the region of interest (Fig 4A and 4B). Two recombinants, H1 and H2, could detect the left marker 11M15.847, while the other two recombinants, H10 and H11, could detect the right marker 11M16.93. Therefore, *qBph11*.*3* was mapped to one region harbored by two markers, 11M15.847 and 11M16.93 (Fig 4C and 4D).

To obtain a high-resolution map *qBph11*.*3*, we screened additional recombinant individuals from the F_3_ population and identified two polymorphic InDel markers, 11M16.781 and 11M16.896, in the target region (Fig 4C). Five recombinants, H3, H4, H6, H7, and H8, defined the left marker 11M16.781, and the other two recombinants, H5 and H9, defined the right marker 11M16.896 (Fig 4D). Gene *qBph11*.*3*, approximately 115 kb according to NIP, was finally mapped in the region between 11M16.781 and 11M16.896. Eleven genes were predicted in the target region: four disease resistance protein genes (*LOC_Os11g29030*, *LOC_Os11g29050*, *LOC_Os11g29090*, *LOC_Os11g29110*), one growth regulator-related protein gene (*LOC_Os11g29120*), two retrotransposon protein genes (*LOC_Os11g29074* and *LOC_Os11g29014*), and four expressed protein genes (*LOC_Os11g29060*, *LOC_Os11g29100*, *LOC_Os11g29130*, and *LOC_Os11g29140*) (S2 Table).

To analyze the candidate genes, we conducted qRT-PCR to validate the four disease-resistance genes in CL48 and KW. *LOC_Os11g29030* and *LOC_Os11g29050* in CL48 cells showed no change in gene expression levels after BPH treatment (Fig 4E and 4F). The expression of *LOC_Os11g29090* was higher in KW than in CL48 at 24 and 48 h (Fig 4G). Moreover, the expression level of *LOC_Os11g29110* detected in CL48 was higher than that of KW (Fig 4H). These results suggest that *LOC_Os11g29090* and *LOC_Os11g29110* may be involved in the resistance of CL48. We sequenced four disease-resistance genes from CL48. Consequently, a 32-bp deletion in the CDS of *LOC_Os11g29090* in CL48 was detected compared to that in NIP and ZH97 (Figs 4I and S4). Moreover, a 1-bp deletion in the CDS of *LOC_Os11g29110* in CL48 was detected compared to that in the NIP (Figs 4J and S5). Several SNP changes were also identified in the CDS of the other two candidate genes between the CL48 and susceptible varieties. However, only a few nonsense SNP exchanges in the genomic sequences of *LOC_Os11g29030* and *LOC_Os11g29050* were detected between CL48 and NIP (S6 and S7 Figs).

### 3.5. MAS and breeding applications of the resistance genes

**Fig 5:**
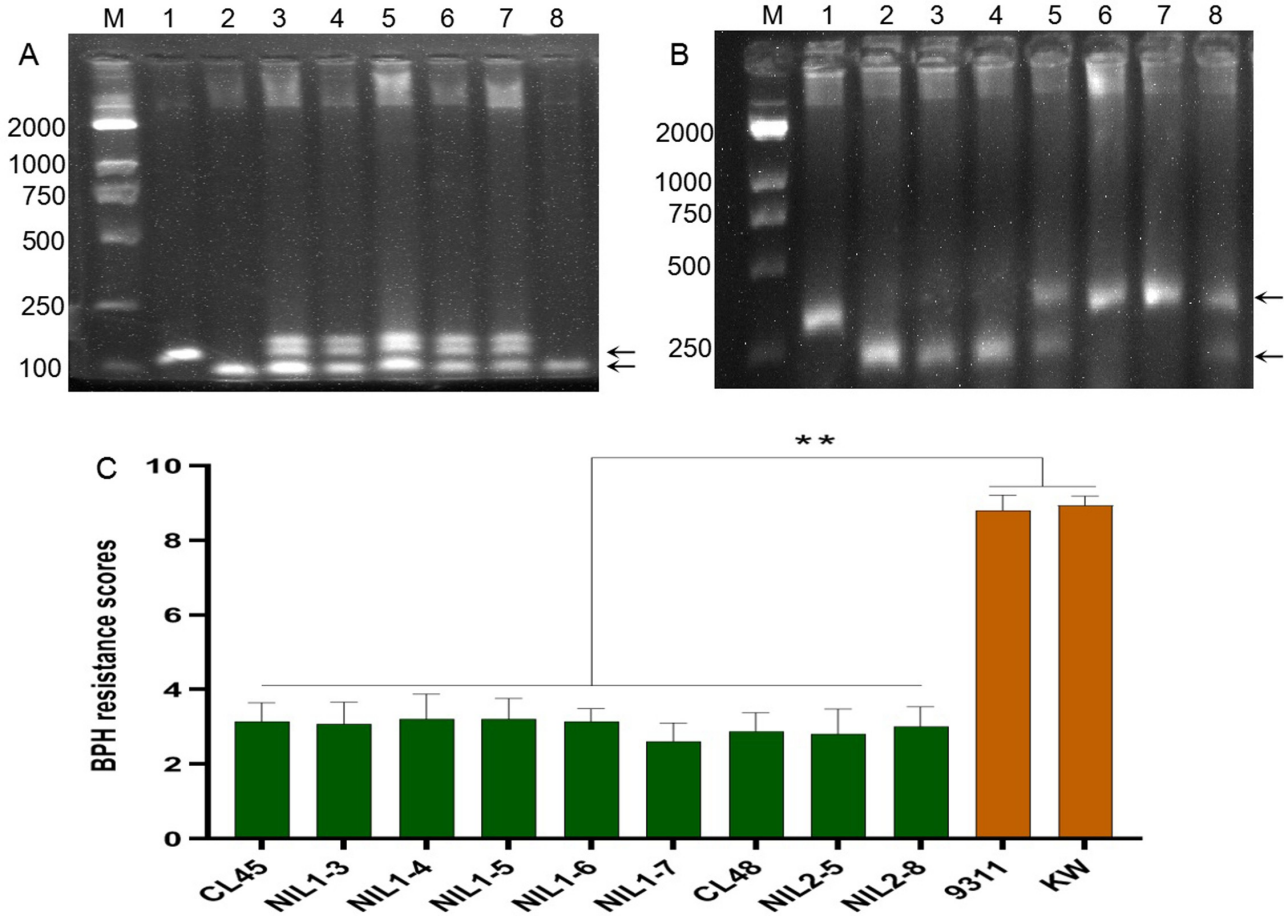
MAS application in NILs. Markers 12M16.983 (A) and 11MA104 (B) detection in 2.5% agarose gel. M, 2000 bp marker, 1–2, CL45 and 9311, 3–8, individuals of NIL1 derived from 9311/CL45 (A) or NIL2 derived from KW/CL48 (B). Arrows on the right show specific fragments of markers 12M16.983 (A) and 11MA104 (B). (C) BPH resistance evaluation of positive NILs at the seedling stage. Lines NIL1-3, NIL1-4, NIL1-5, NIL1-6, and NIL1-7 in (C) were respectively derived from individuals of 3, 4, 5, 6, and 7 in (A); Lines NIL2-5 and NIL2-8 were respectively derived from individuals of 5 and 8 in (B). Error bars represent the SD. *, P < 0.05; **, P < 0.01.

Two markers, 12M16.983 and 11MA104, were developed for the resistance genes CL45 and CL48 to apply MAS. Specifically, marker 12M16.983 yielded one band of 170 bp when the PCR product was electrophoretically displayed on an agarose gel. On contrary, only one band measuring approximately 130 bp was detected in the susceptible varieties (Fig 5A, lanes 1 and 2; and S8 Fig). The InDel marker 11MA104 had an insertion of 104 bp in CL48 compared to NIP and other susceptible varieties. This yielded one band of approximately 270 bp in CL48, whereas the susceptible varieties had one band of about 170 bp (Fig 5B, lanes 1 and 2; S9 Fig). Five individuals positive for BC_3_F_1_ from 9311/CL45 and two individuals positive for BC_4_F_1_ from KW/CL48 were selected for BPH resistance identification, and all showed a highly resistant phenotype (Fig 5C). These results indicate that both markers could be used for MAS.

Line 9311 is an elite restorer line for hybrid rice, and KW is a widely cultivated rice variety in South China. To improve BPH resistance, they were crossed with the resistant accessions CL45 and CL48. Positive backcrossing progenies were selected using the tightly linked markers 12M16.983 in CL45 and 11MA104 in CL48.

**Fig 6:**
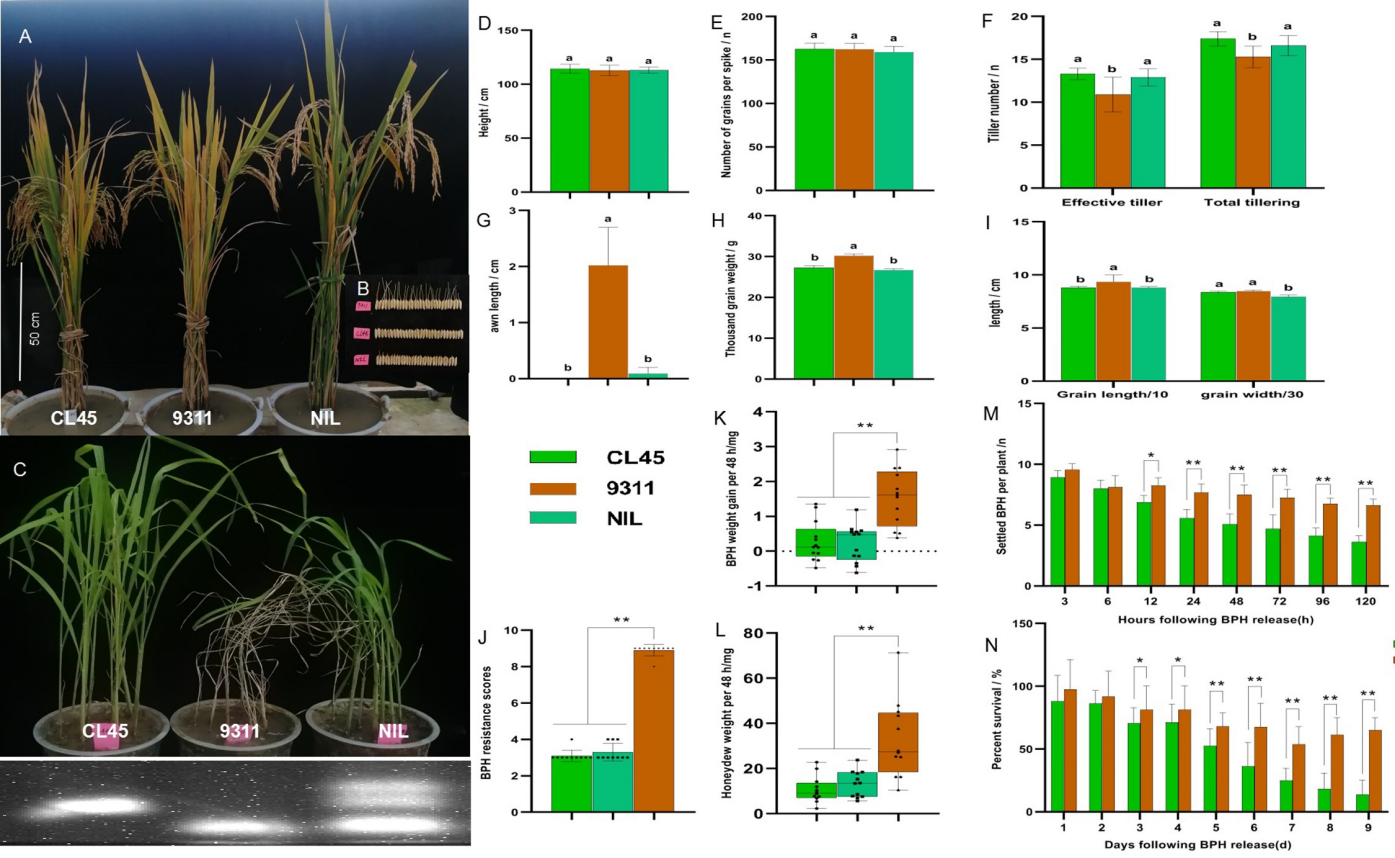
Phenotype of agronomic traits and BPH resistance of NIL1. (A) Phenotype of plant height, effective tillering, and total tillering of CL45, 9311, and NIL1 at maturation stage, bar = 50 cm. (B) Phenotype of awn length, grains length, and grains width of CL45, 9311, and NIL1 at maturation stage, bar = 1 cm. (C) Phenotype of seedling resistance tests of CL45, 9311, and NIL1. The PCR band patterns amplified with marker 12M16.983 showed CL45 and NIL1 with resistance gene, and 9311 contained no resistance gene. (D) Plant height; (E) Number of per spike; (F) Effective tillering and total tillering; (G) Awn length; (H) Thousand-grain weight; (I) Grains length and width of CL45, 9311, and NIL1 at maturation stage. Bars represent the means of nine replicates. Error bars represent the SD. Means labeled with the same letter are not significantly different at a level of P = 0.05. (J) BPH-resistance scores of CL45, 9311, and NIL1 at seedling stage. (K) Weight gains; and (L) Honeydew excretion of BPH of CL45, 9311, and NIL1 at the tillering stage. Detection of (M) BPH host choice test and (N) BPH survival rates on CL45, 9311, and NIL1.Bars represent means of eight replicates. Error bars represent the SD. *, P < 0.05; **, P < 0.01.

The 9311/CL45 lines were backcrossed four times with 9311 and self-crossed three times consecutively, resulting in NIL1. The awn length, grain length, thousand-grain weight, and other agronomic traits were measured. The effective tillering, total tillering, thousand-grain weight and grains length of NIL1 were mainly consistent with those of CL45, especially the awn length (Fig 6A, 6B, 6F, 6G, 6H and 6I). The grain width of NIL1 was consistent with that of 9311 and significantly different from that of CL45 (Fig 6B and 6I). However, plant height and number per spike of NIL1 were not significantly different between the two parents (Fig 6A,6D and 6E). NIL1 had a resistance value of 3.4 during the seedling stage (Fig 6C and 6J). During the tillering stage, the average body weight gain and honeydew weight excreted by a single BPH after 48 h fed with NIL1, the number of BPH distributed on NIL1 in the BPH host choice test after 12 h infestation, and the BPH survival rates of NIL1 after two days of infestation were significantly lower than those of 9311 (Fig 6K–6N). These results indicated that BPH growth and development were inhibited in NIL1 plants, similar to CL45.

**Fig 7:**
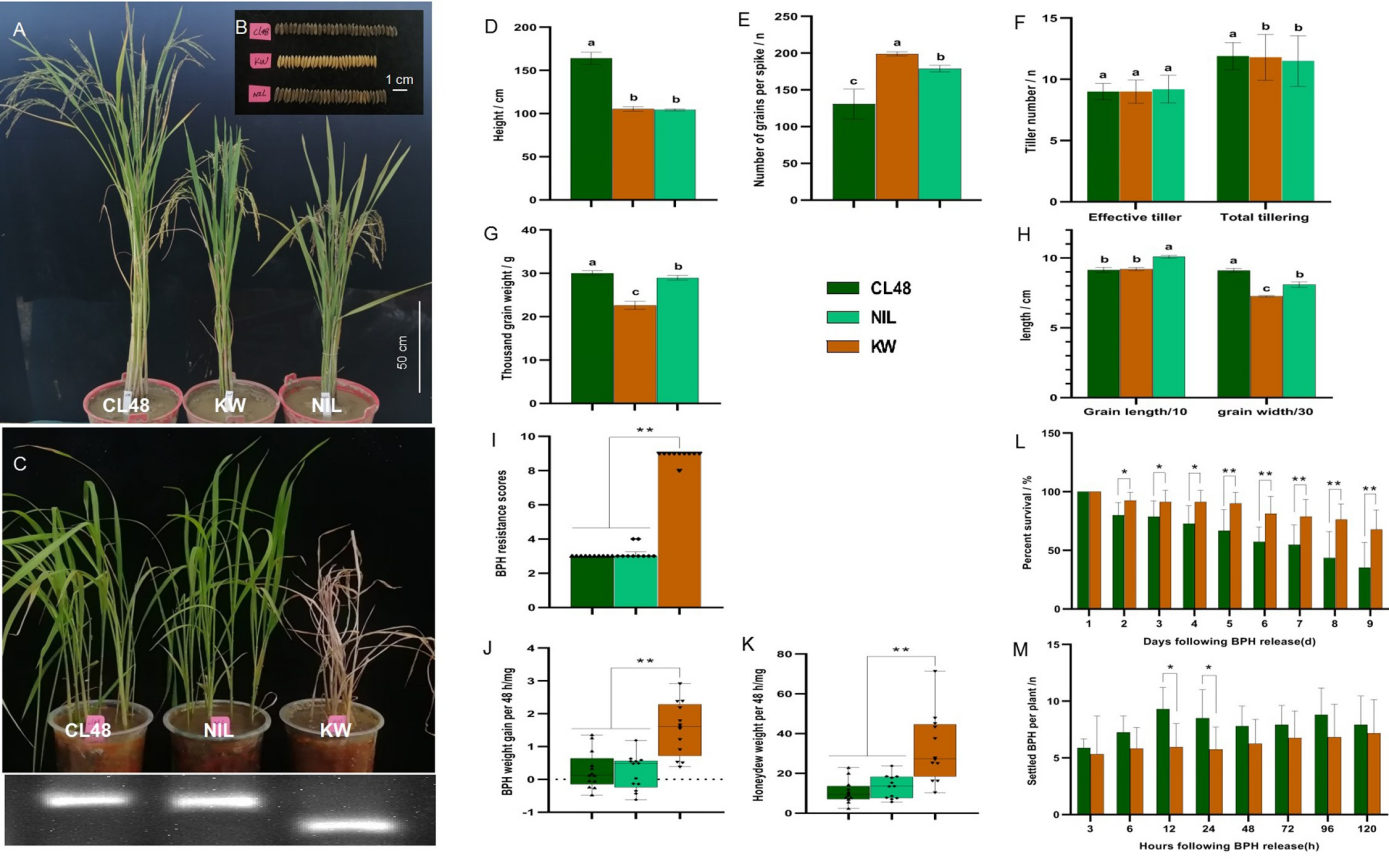
Phenotype of agronomic traits and BPH resistance of NIL2. (A) Phenotype of plant height, effective tillering, and total tillering of CL48, KW, and NIL2 at maturation stage, bar = 50 cm. (B) Phenotype of grains length and grains width of CL48, KW, and NIL2 at maturation stage, bar = 1 cm. (C) Phenotype of seedling resistance tests of CL48, KW, and NIL2. The PCR band patterns amplified with marker 11MA104 showed CL48 and NIL2 with resistance genes, and KW contained no resistance genes. (D) Plant height; (E) Number of per spike; (F) Effective tillering and total tillering; (G) Thousand-grain weight; (H) Grains length and width of CL48, KW, and NIL2 at maturation stage. Bars represent the means of nine replicates. Error bars represent the SD. Means labeled with the same letter are not significantly different at a level of P = 0.05. (I) BPH-resistance scores of CL48, KW, and NIL2 at seedling stage. (J) Weight gains; and (K) Honeydew excretion of BPH of CL48, KW, and NIL2 at the tillering stage. Detection of (L) BPH host choice test and (M) BPH survival rates on CL48, KW, and NIL2. Bars represent means of eight replicates. Error bars represent the SD. *, P < 0.05; **, P < 0.01.

KW/CL48 was backcrossed four times with KW and self-crossed three times, resulting in the formation of NIL2. The plant height and total tiller number of NIL2 were mainly consistent with those of KW; in particular, the plant height was dwarfed from an average of 164 cm for the parent CL48 to an average of 105 cm for KW (Fig 7A, 7D and 7F). The difference was significant, and there was no lodging phenomenon, as in parent CL48, during the heading and wax ripening stages. The grain number per spike, thousand-grain weight, grain length and grain width of NIL2 were significantly different from the two parents, between CL48 and KW (Fig 7A, 7B, 7E, 7G and 7H). The effective tiller number of NIL2 was not different from two parents (Fig 7A and 7F). NIL2 had a resistance value of 3.2 during the seedling stage (Fig 7C and 7I). During the tillering stage of NIL2, the average body weight gain and honeydew weight excreted, host choice, and survival rates of BPH were consistent with those of CL48 and significantly different from those of 9311 (Fig 7J–7M). At the same time, it was also found that the positive individuals selected with the 11MA104 marker maintained the same black glume color as CL48, while the individuals with black glume color also showed positive identification with the 11MA104 marker. They exhibited significantly higher resistance against BPHs, such as CL48, indicating that the black glume color in CL48 is closely linked to the BPH resistance gene and can be effectively utilized in breeding.

**Fig 8:**
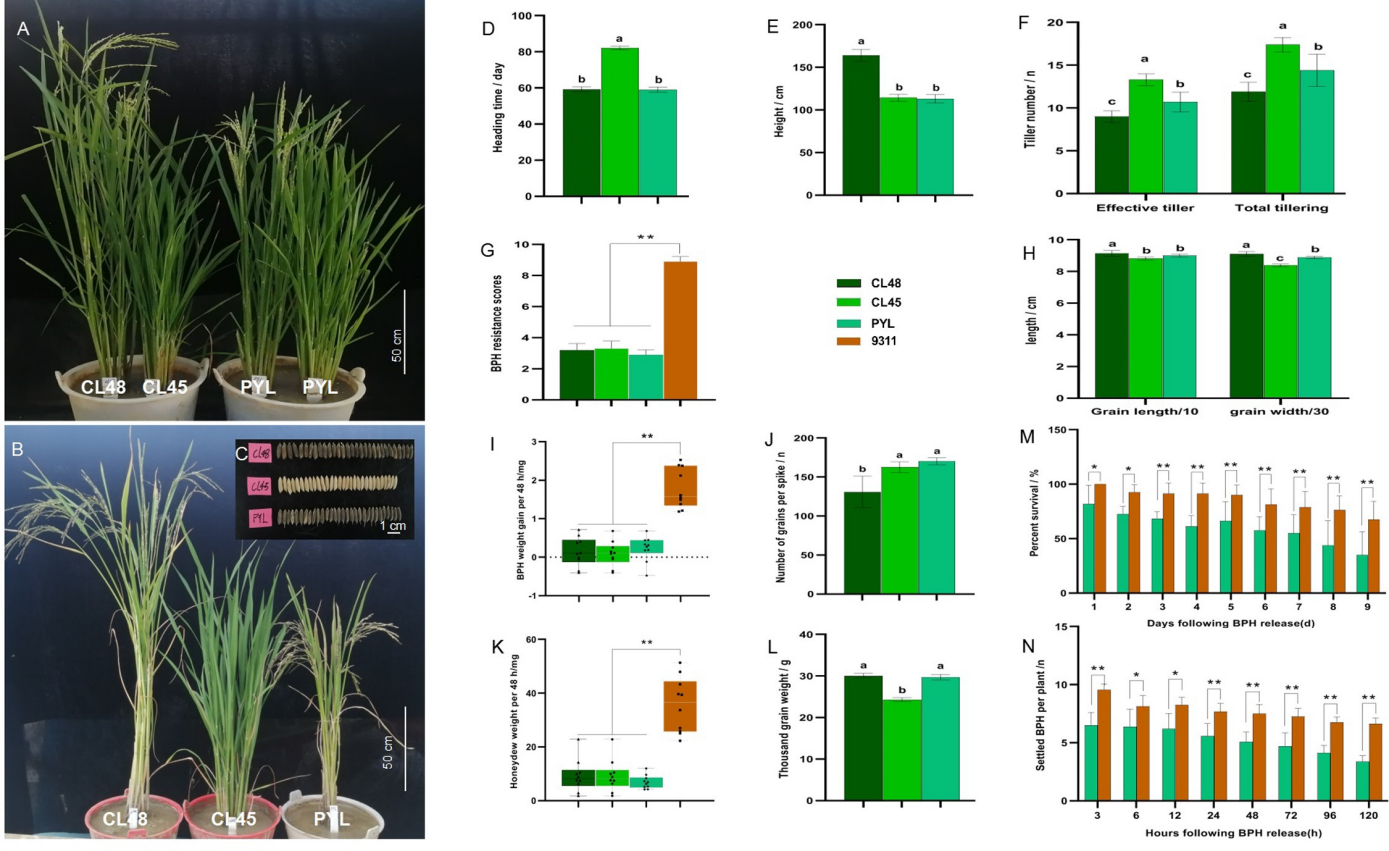
Phenotype of agronomic traits and BPH resistance of PYLs. (A) Phenotype of heading time of CL48, CL45, and PYL. Bar = 50 cm. (B) Phenotype of plant height, effective tillering, and total tillering of CL48, CL45, and PYL at maturation stage, bar = 50 cm. (C) Phenotype of grains length and grains width of CL48, CL45, and PYL at maturation stage, bar = 1 cm. (D) Heading time; (E) Plant height; (F) Effective tillering and total tillering at maturation stage. (G) BPH-resistance scores of CL48, CL45, and PYL at the seedling stage. (H) Grains length and width at maturation stage. (I) Weight gains of BPH of CL48, CL45, and PYL at the tillering stage. (J) Number of per spike at maturation stage. (K) Honeydew excretion of BPH of CL48, CL45, and PYL at the tillering stage. (L) Thousand-grain weight of CL48, CL45, and PYL at maturation stage. Detection of (M) BPH host choice test and (N) BPH survival rates on CL48, KW, and PYL. Bars represent the means of nine replicates for (D, E, F, H, J, and L), eight replicates for (G, I, K, M, and N). Error bars represent the SD. Means labeled with the same letter are not significantly different at a level of P = 0.05. *, P < 0.05; **, P < 0.01.

As the four parents had many differences in agronomic traits, especially the resistant parents CL45 and CL48, the pyramided F_1_ individuals were continuously self-pollinated five times to obtain the F_6_ generation, named PYLs. Only plants with comprehensive elite agronomic traits were selected for the next generation during this process. The heading time and thousand-grain weight of PYLs were consistent with those of CL48 but significantly different from those of CL45 (Fig 8A–8D and 8L). The PYLs heading time was shortened by approximately 25 days, from 84 to 58 days. The plant height, grain length, and grain number per spike of PYLs were consistent with those of CL45 but significantly different from those of CL48 (Fig 8B, 8C, 8E, 8H and 8J). In particular, the PYLs plant height ranged from 164 to 110 cm as CL45, and the dwarf was approximately 50 cm. Other traits, such as effective tillering, total tillering, and grain width, differed between the two parents (Fig 8B, 8C, 8F and 8H). The PYLs showed high resistance to BPH at the seedling and tillering stage (Figs 8G, 8I, 8K, 8M, 8N, S10A and S10B) and significantly inhibited the growth and development of BPHs during the tillering stage (average body weight gain and honeydew weight excreted by BPH, host choice, and survival rates were significantly lower than those of the two parents). At the same time, the glume color of the PYLs was consistent with that of CL48, which was significantly different from that of CL45. This can assist in the selection of positive individuals carrying the CL48 gene. Combined with the 12M16.983 marker of CL45, it could effectively select the positive individuals for the two resistant materials.

## 4. Discussion

BPH is the most destructive pest of rice, resulting in a significant annual decrease in yield [26, 27]. Approximately 45 BPH resistance genes have been identified, 12 of which have been cloned. In the present study, *Bph46* was finely mapped to chromosome 12 and flanked by markers SNP-5 and SNP-6 (Fig 4). Ten BPH resistance genes *(Bph1*, *bph2*, *bph7*, *Bph9*, *Bph10*, *Bph16*, *Bph18(t)*, *Bph19(t)*, *Bph21(t)*, and *Bph26*) are located to the right of *Bph46* on the long arm of chromosome 12 [28], among which *bph7* is the closest according to the manufacturer’s information. Therefore, it is considered a novel locus/gene as no BPH resistance genes have been previously reported in the target region. After conducting qRT-PCR and sequencing of candidate genes in CL45, *Gene 3* was expressed after BPH treatment. Additionally, a 6-bp SNP deletion in the 5’-UTR regions and some SNP changes, were noted. Cloning and functional analyses will be performed to verify this hypothesis. Gene *qBhp11*.*3*, derived from CL48, was located in a 145-kb region on chromosome 11 and harbored markers 11M16.751 and 11M16.896 (Fig 5). *Bph28(t)* from variety DV85 was physically defined to an interval of 64.8 kb on chromosome 11 and located on the right of *qBhp11*.*3* [29]. *Bph43* from variety IRGC8678 was also described as an interval of 280 kb on chromosome 11 according to the NIP genome sequence [30]. According to the markers and reference genome sequence, Gene *qBph11*.*3* is included in the region where *Bph43* is located. However, it is still difficult to determine whether both genes are the same or allelic, because the mapping region of *Bph43* is large. Further gene transformation and insect resistance evaluations will confirm this hypothesis.

A cluster of four disease resistance genes in the fine mapping interval of CL48 may represent a sophisticated defense mechanism evolved by plants to ward off diverse pathogens or insects. For instance, at least 38 NLR genes were detected in one 1.35-Mb region at the end of chromosome 11 according to the NIP genome, with resistance genes for rice bacterial blight (*Xa3*/*Xa26*) and rice blast *Pikml*/*Pikm2* [31–33]. *Bph3* from Rathu Heenati, consisting of three lectin receptor kinase genes *OsLecRK1*-*OsLecRK3*, is located on the plasma membrane and collaborates to offer extensive and long-lasting insect resistance in rice [4]. qRT-PCR analysis indicated that *LOC_Os11g29090* and *LOC_Os11g29110* showed significant differences in expression levels between CL48 and KW after BPH treatment. Moreover, genomic and CDS sequence analyses indicated larger changes in both genes between the resistant and susceptible varieties. These results suggest that they may be involved in the resistance of CL48.

Since most NLR genes are specific to pathogens or insect resistance but are not durable, the pesticide has been promoted on a large scale. New ‘biological’ diseases or BPHs are likely to continue to emerge. The gradual loss of resistance in rice varieties ‘IR26’ with *Bph1* and ‘IR36’ with *Bph2* was an obvious example [25, 34]. Therefore, the most efficient and cost-effective way to control BPH damage is to explore new resistant germplasms and breed insect-resistant varieties using MAS. In this study, two simple and effective tightly linked markers, 12M16.983 and 11MA104, were developed for genes derived from CL45 and CL48, respectively. Furthermore, several highly BPH-resistant NILs with 9311 or KW genetic backgrounds, as well as PLYs with two resistant parents, were developed with MAS. These results could contribute to the development of an insect-resistant rice breeding program. These results indicated that the MAS method is an effective and efficient way to develop BPH resistance varieties. Interestingly, we found that the black glume color is closely linked to the BPH resistance gene in CL48 and can effectively assist in identification of positive individuals in breeding. The application of CL45 and CL48 in NILs and PYLs breeding for resistance to BPH can effectively improve the agronomic traits of recurrent or resistant parents, such as plant height, panicle stage, effective tillering, and thousand-grain weight. Most importantly, it significantly enhanced the resistance of rice to BPH, which is of great significance for insect-resistant rice breeding programs and safe rice production.

## 5. Conclusion

1. *Bph46* is a novel BPH resistance gene derived from the rice landrace accession CL45, which is finely mapped to a 450 kb region on chromosome 12. NIL1 carrying *Bph46* was developed using the specific marker 12M16.983 and exhibited significantly high resistance against BPH.
2. The resistance gene derived from the rice landrace accession CL48 was finely mapped to an interval of 145 kb on chromosome 11. Sequence comparison and qRT-PCR analysis suggested that *LOC_Os11g29090* and *LOC_Os11g29110* were the most likely resistance genes. NIL2 containing *qBph11*.*3* was developed using the specific marker 11MA104 and exhibited significantly high resistance against BPH. Interestingly, the black glume color is tightly linked to *qBph11*.*3* and can be effectively used to select resistant plants in breeding programs.
3. PYLs carrying both BPH resistance genes were developed with MAS, which showed significantly higher resistance against BPHs.

### 5.1. Plant guideline statement

The appropriate permissions and/or licenses for the collection of plant or seed specimens. The variety of Landrace rice accessions CL45 and CL48 used in this experiment were selected and cultivated by the project host, Professor Qiu Yongfu in 2019, and we received permission, support, and guidance from Professor Qiu Yongfu throughout the experiment.

In the meantime, experimental research and field studies on plants (either cultivated or wild), including the collection of plant material, we comply with the IUCN Policy Statement on Research Involving Species at Risk of Extinction and the Convention on the Trade in Endangered Species of Wild Fauna and Flora.

## Supporting information

S1 Fig BPH resistance of CL45 and CL48 at seedling and tillering stage. (TIF)

S2 Fig Cultivation of near-isogenic lines (NILs) and pyramided lines (PYLs). (TIF)

S3 Fig CDS comparison of *Gene 3* between CL45 and MH63. (TIF)

S4 Fig CDS compared of *LOC_Os11g29090*. (TIF)

S5 Fig CDS compared of *LOC_Os11g29110*. (TIF)

S6 Fig Gene sequence compared of *LOC_Os11g29030*. (TIF)

S7 Fig Gene sequence compared of *LOC_Os11g29050*. (TIF)

S8 Fig Markers 12M16.983 detection in 2.5% agarose gel. (TIF)

S9 Fig Markers 11MA104 detection in 2.5% agarose gel. (TIF)

S10 Fig BPH resistance of PYL at seedling and tillering stage. (TIF)

S1 Table Predicted candidate genes for *Bph46* in Nipponbare reference genome. (XLSX)

S2 Table Predicted candidate genes for *qBph11*.*3* in Nipponbare reference genome. (XLSX)

S3 Table The primers used in this study. (XLSX)
