# Supplementary figures and images for: Fine mapping and breeding application of two brown planthopper resistance genes derived from landrace rice
Source: PLoS One. 2024 Apr 16;19(4):e0297945. doi: 10.1371/journal.pone.0297945 (PMC11020626; doi:10.1371/journal.pone.0297945)

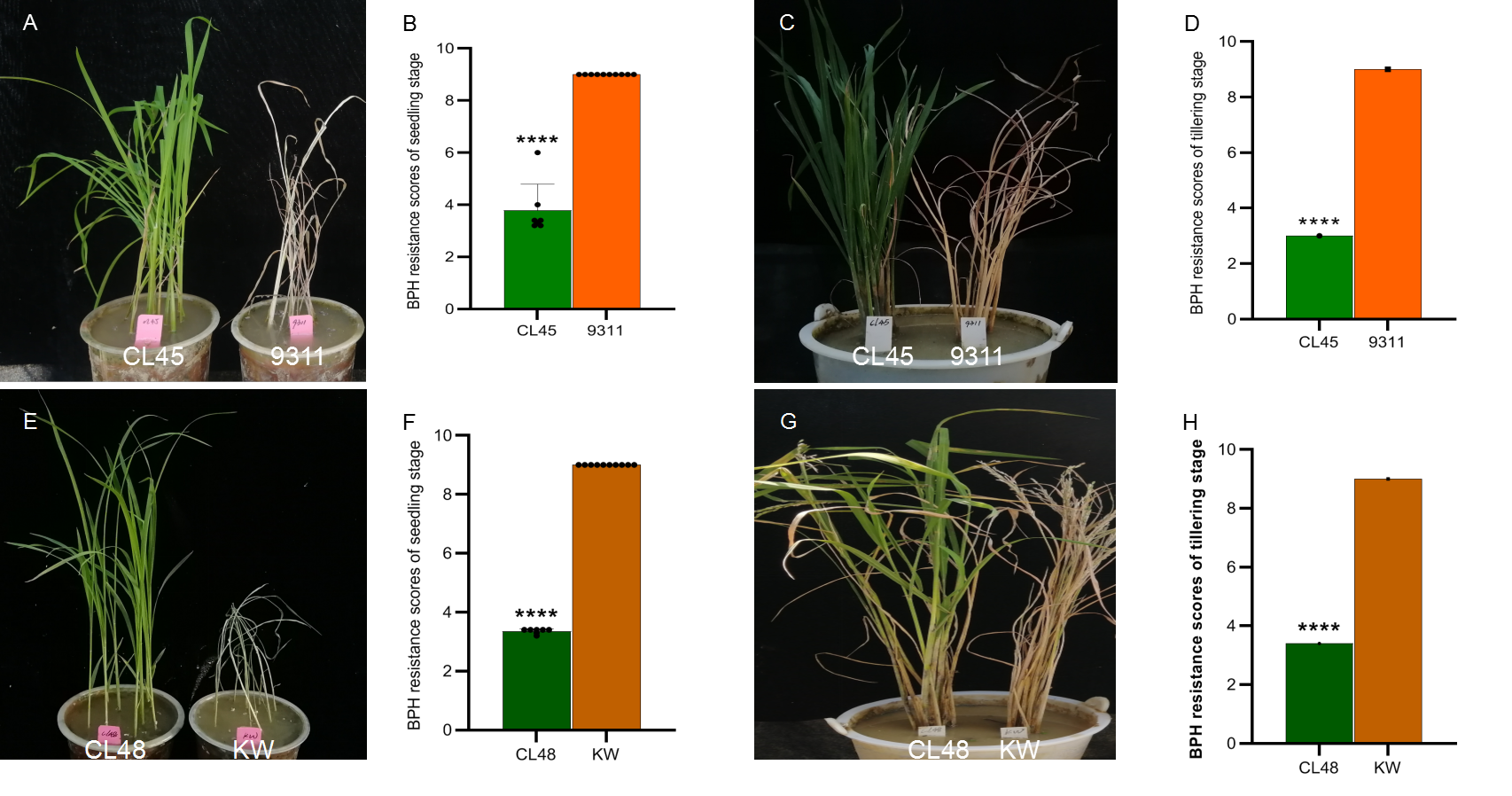

Supplement: S1 Fig — (TIF) [file pone.0297945.s001.tif]

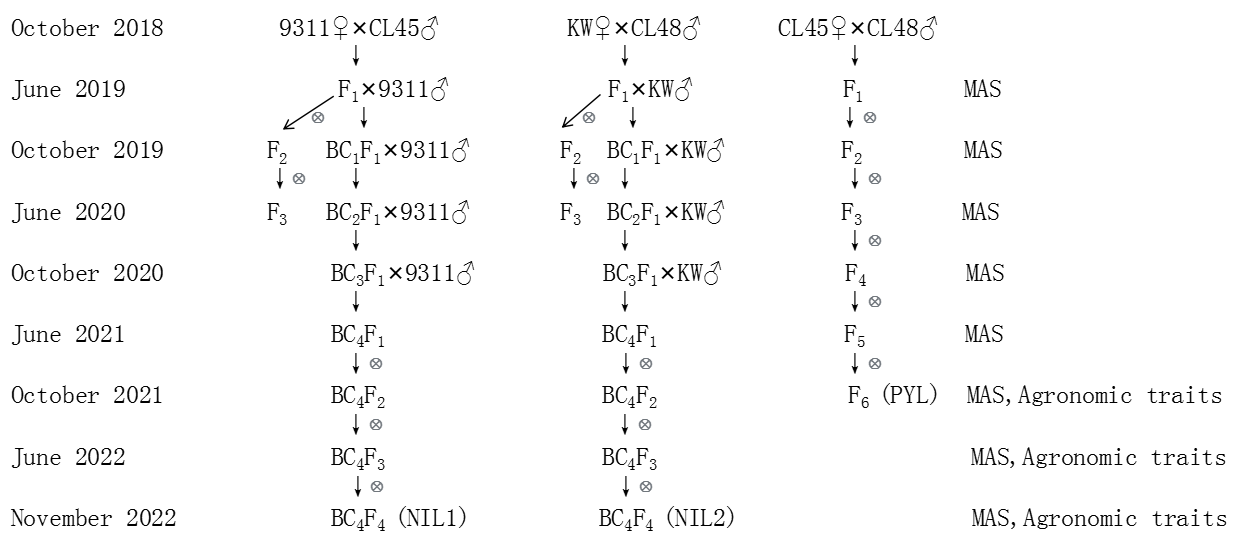

Supplement: S2 Fig — (TIF) [file pone.0297945.s002.tif]

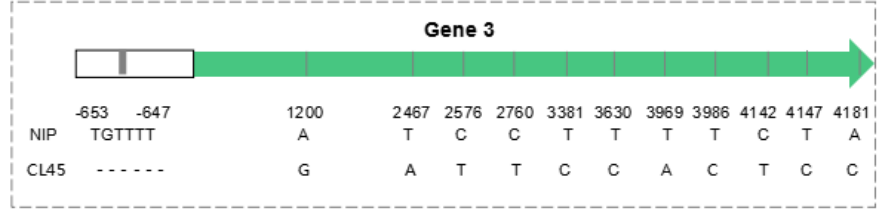

Supplement: S3 Fig — (TIF) [file pone.0297945.s003.tif]

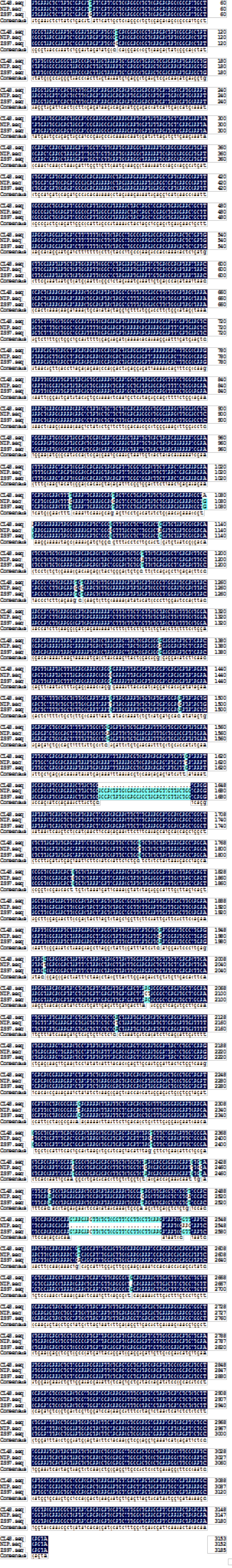

Supplement: S4 Fig — (TIF) [file pone.0297945.s004.tif]

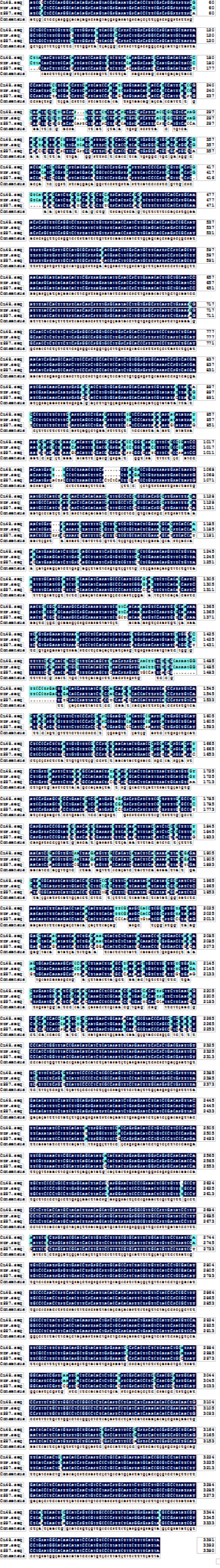

Supplement: S5 Fig — (TIF) [file pone.0297945.s005.tif]

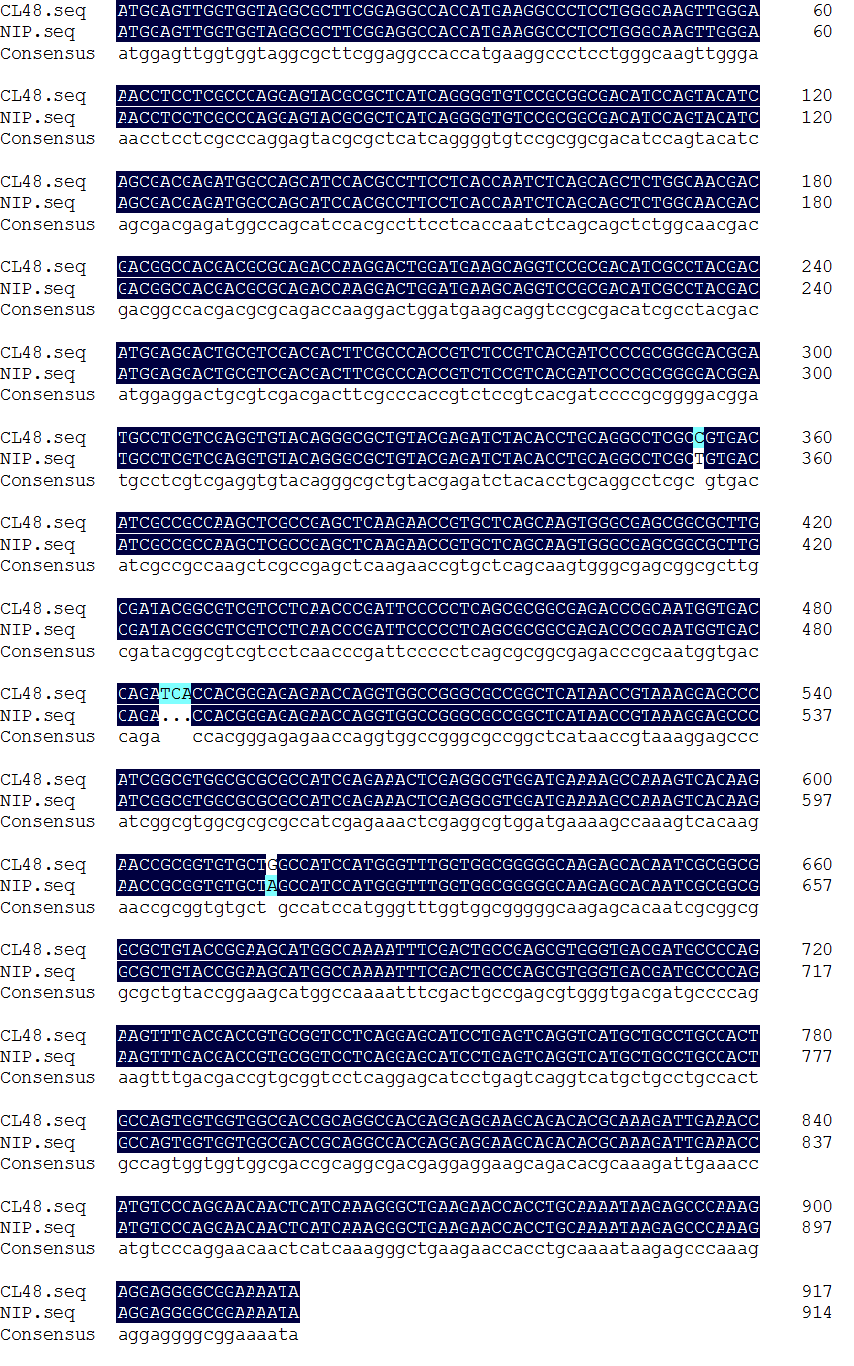

Supplement: S6 Fig — (TIF) [file pone.0297945.s006.tif]

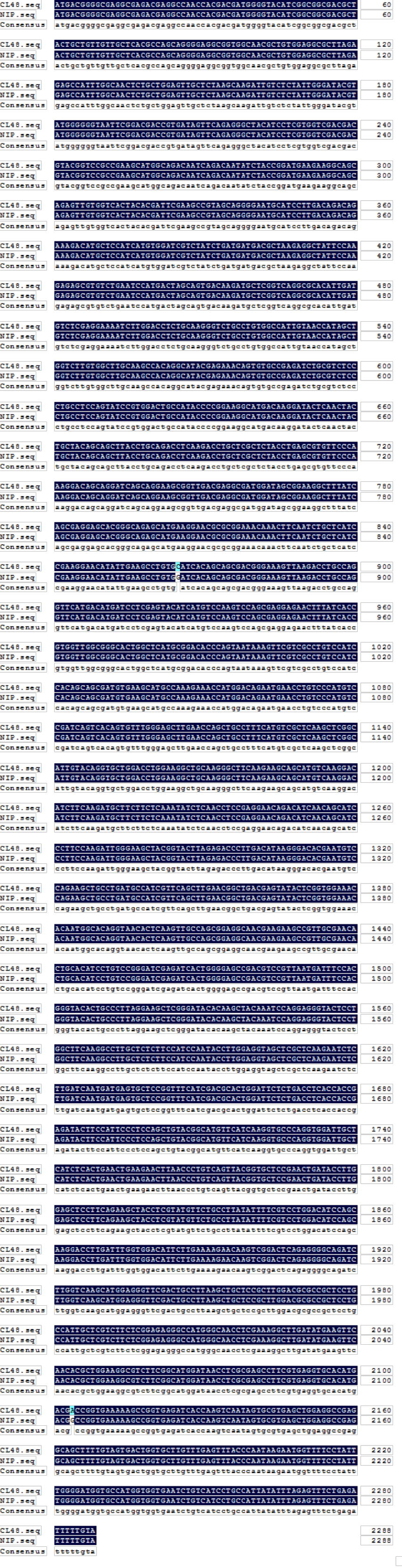

Supplement: S7 Fig — (TIF) [file pone.0297945.s007.tif]

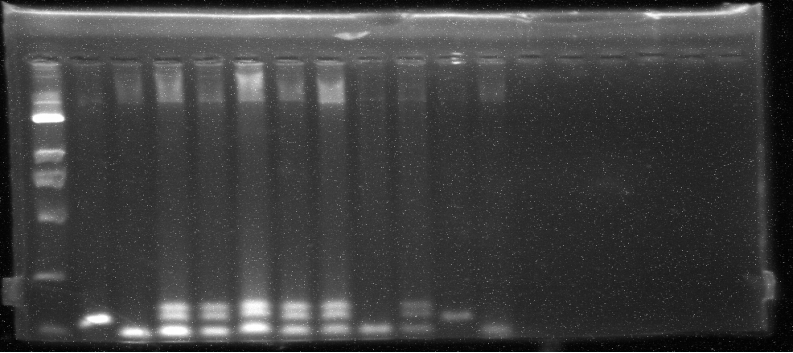

Supplement: S8 Fig — (TIF) [file pone.0297945.s008.tif]

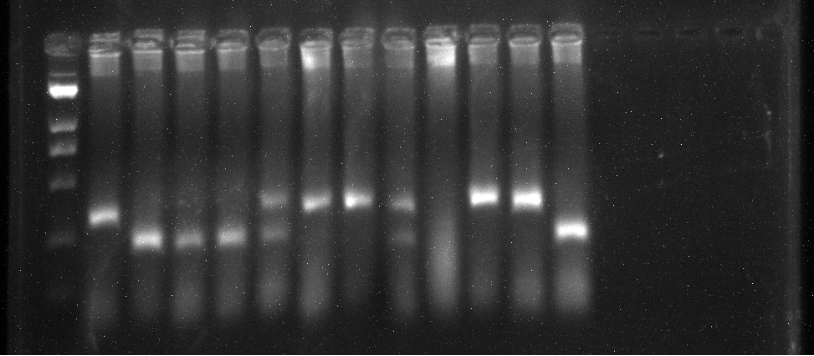

Supplement: S9 Fig — (TIF) [file pone.0297945.s009.tif]

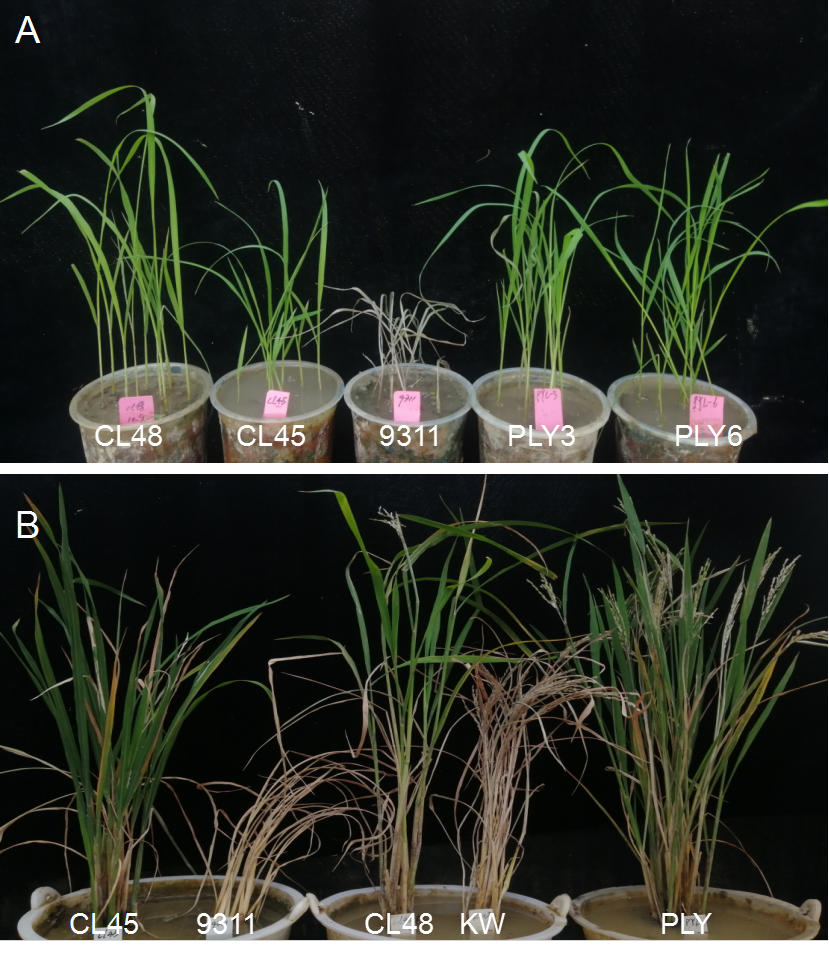

Supplement: S10 Fig — (TIF) [file pone.0297945.s010.tif]
